# Effects of Caloric Restriction on Spatial Object Recognition Memory, Hippocampal Neuron Loss and Neuroinflammation in Aged Rats

**DOI:** 10.3390/nu15071572

**Published:** 2023-03-24

**Authors:** Marta Portero-Tresserra, Neus Galofré-López, Elisabet Pallares, Claudia Gimenez-Montes, Carlos Barcia, Roser Granero, Divka Rojic-Becker, Anna Vale-Martínez, Margarita Martí-Nicolovius, Gemma Guillazo-Blanch

**Affiliations:** 1 Departament de Psicobiologia i Metodologia de les Ciències de la Salut, Institut de Neurociències, Universitat Autònoma de Barcelona, 08193 Barcelona, Spain; 2 Departament de Bioquímica i Biologia Molecular, Institut de Neurociències, Universitat Autònoma de Barcelona, 08193 Barcelona, Spain; 3 Ciber Fisiopatología Obesidad y Nutrición (CIBERObn), Instituto de Salud Carlos III, 28029 Madrid, Spain; 4 Psychoneurobiology of Eating and Addictive Behaviors Group, Neurosciences Programme, Bellvitge Institute for Biomedical Research (IDIBELL), 08908 Barcelona, Spain

**Keywords:** aging, spatial memory, hippocampus, neurogenesis, microglia

## Abstract

Age-related neurobiological changes significantly affect hippocampal structure and function, such that the main cognitive impairments associated with aging are related to the integrity of this brain structure, including the deterioration in spatial object recognition (SOR) memory. Previous studies have shown that intrinsic factors such as neuroinflammation, as well as lifestyle factors such as diet, can affect aging-associated brain functions and cognitive performance. In this regard, caloric restriction (CR) produces beneficial effects on health and life expectancy, although its ability to slow down age-dependent effects on cognitive decline and hippocampus (HPC) functioning remains unclear. Therefore, we set out to evaluate the effects of CR on SOR memory in aged male Wistar rats, as well as those on hippocampal neuron loss, neurogenesis and inflammation. The data show that CR in aged rats attenuates the decline in SOR memory, age-associated hippocampal neuron loss, and age-dependent microglial activation. Furthermore, we found a significant reduction in neurogenesis in the dentate gyrus of the old animals relative to adult rats. These findings support the positive effect of CR on SOR memory, suggesting that it dampens hippocampal neuronal loss and reduces proinflammatory activity.

## 1. Introduction

Aging can be defined as progressive, physiological and functional deterioration over the adult life of an individual [1], and it is the result of complex interactions of genes and environmental factors that eventually enhance susceptibility to disease or death [1,2,3]. Both pathological and normal aging are characterized by impaired cognitive performance, particularly in terms of memory formation and consolidation, as well as various neurobiological modifications [2,4].

Inflammaging is the low-grade, slow, chronic upregulation of pro-inflammatory responses, and it is one of the progressive neurobiological modifications that occur in the aging brain [5,6]. This age-related enhancement of chronic systemic inflammatory status is particularly evident in the hippocampus (HPC), a brain region involved in explicit memory consolidation, and in spatial and context-dependent learning [2,3,7]. Moreover, the HPC displays numerous electrophysiological, structural and morphological changes during normal aging. In addition to inflammation, the age-related hippocampal alterations include changes in volume and synaptic plasticity, as well as a dampening of neurogenesis [8,9,10]. In fact, aging is a well-known negative regulator of neurogenesis [11], with a decline in this process reported in aged rodents [12] and non-human primates [13]. Adult hippocampal neurogenesis (AHN) is a form of ongoing plasticity that occurs throughout life and it involves the birth, differentiation and maturation of new neurons in the mammalian dentate gyrus (DG). Since neurogenesis seems to be important in maintaining cognitive processes such as memory consolidation and emotional behaviour, this reduction in the production of neurons could be correlated with age-dependent hippocampal learning and memory impairment [10,14]. Interestingly, numerous studies have identified neurogenesis as a very plastic process regulated by a variety of physiological, environmental and pathological factors [11,15]. Indeed, an increase in pro-inflammatory cytokines and/or reduced liberation of growth factors by hippocampal astrocytes seem to be causal factors in the age-related decline in neurogenesis [2].

Aging is tightly linked to microglial cell activation, the cells that constitute the first barrier of the innate immune system in the brain and which are critical elements in maintaining the integrity of the nervous system [16]. However, microglial dysregulation can also lead to excessive or chronic neuroinflammation [2] and, as a consequence, brain damage or neurological disease [17]. A high concentration of microglial cells can be found in the aged HPC [18,19]. The release of cytokines as part of the pro-inflammatory response, such as tumour necrosis factor- α (TNF-α), interleukin 6 (IL-6), and interleukin 1β (IL-1β), is likely to inhibit the differentiation of neural precursor cells (NPCs) and favour the production of astrocytes [20]. In addition, microglia with a neurotoxic phenotype are considered to participate in the phagocytosis of new-born neurons that fail to integrate into existing circuits, regulating glutamatergic receptor maturation and synaptic transmission, and supporting synaptic pruning [21]. In line with this, the macrophage/microglial ionized calcium-binding adaptor 1 (Iba-1) marker was significantly elevated in the HPC of aged animals compared to adults [22].

Intrinsic factors such as neuroinflammation, oxidative stress and brain damage, as well as lifestyle factors such as diet, seem to affect adult neurogenesis [23] and aging-associated brain functions. In this sense, many studies have shown that a caloric restricted (CR) diet significantly extends lifespan [24], ameliorating brain aging by activating anti-inflammatory responses, promoting neurogenesis, and enhancing monoaminergic and glutamatergic neurotransmission in the HPC [4,25,26,27]. Nevertheless, the ability of CR to protect against the cognitive decline that accompanies aging remains unclear [28,29]. Since microglial activation, along with changes in HPC volume and neurogenesis, may be correlated with cognitive decline in aged rats, we herein evaluate the capacity of CR to prevent age-dependent hippocampal-related memory decline and neuroinflammation, and its capacity to preserve the existing HPC functions.

## 2. Materials and Methods

### 2.1. Subjects

**Figure 1:**
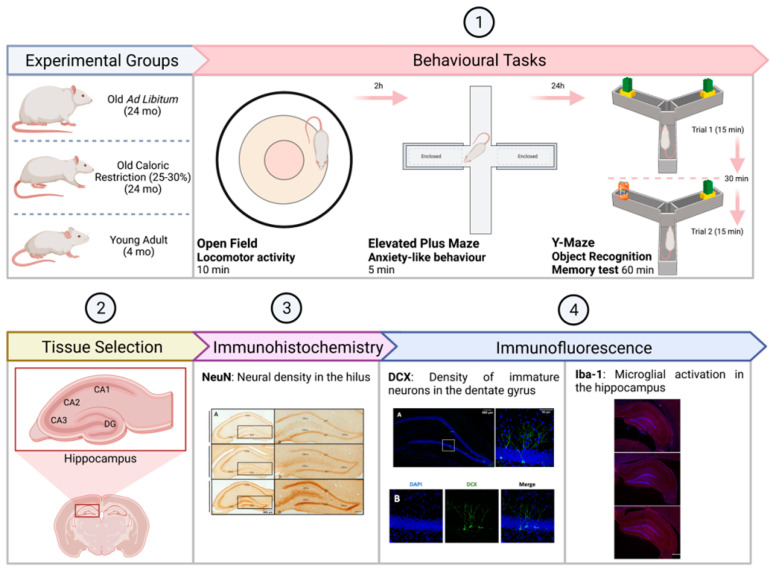
Overview of the experimental design (created with BioRender.com). OLD AL: Old AdLibitum group; OLD CR: Old Caloric Restriction group; YA: Young Adult group; EPM: elevated plus maze; OF: Open field; SOR: spatial object-recognition.

Male Wistar rats (n = 34: Prolabor, Charles River Laboratories, Abresle, France) were obtained from the breeding stock at the Psychobiology and Methodology of Health Sciences Laboratory (Universitat Autònoma de Barcelona) and they were assigned randomly to one of three experimental groups: a Young Adult (YA) group (n = 12; age = 3–4 months); a Caloric Restriction (CR) group (n = 12; age = 24–27 months); and an Ad Libitum (AL) group (n = 10; age = 24–27 months). The mean body weight of the rats in each group was: YA, 481.48 ± 9.7 g; CR, 502.52 ± 8.48 g; and AL, 667.85 ± 20.30 g. The CR group followed a food restriction protocol [30] that involved a 25–30% reduction in food intake from four months of age [4,31], whereas the AL group was composed of old rats that had unrestricted access to food. All animals were fed on standard laboratory food of dry pellets (obtained from Harlan Laboratories Inc., Madison, USA) that was made available once daily at 8 am, and all groups had free access to water. All the animals were paired and housed in 50 × 22 × 14 cm transparent plastic cages with sawdust bedding, and they were maintained in a controlled environment of 60–70% humidity, a temperature of 20 to 22 °C, and on a 12-h light–dark cycle [31]. All the procedures were performed following the EU Directive for the protection of animals used for experimental and other scientific purposes (2010/63/EU), and with the authorization of the Generalitat de Catalunya (DOGC 2450 7/8/1997, DARP number 3866). A summary of the experimental design is presented in Figure 1.

### 2.2. Measures

#### 2.2.1. Behavioural Tasks

**Open Field (OF).** Open field is a sensorimotor task which allows the evaluation of general locomotor activity levels and the capacity to undertake exploration of the space, and it also measures the basal levels of anxiety in rodents. The specific procedure used in the OF test was based according to an earlier method [4,31]. The OF test was a circular arena with walls to prevent escape (80 cm in diameter and 34 cm high walls). The animal performance in each session was recorded with a camera (JVC, Barcelona, Spain, Everio Model GZ-X900) and the variables were analysed with a computer software (Smart Video Tracking System, Version 3, Panlab, Barcelona, Spain). The field was split into three different zones (centre, medium and peripheric), and each animal was placed in the periphery area facing the wall at the beginning of the test and then allowed to move freely for 10 min. The variables of permanence in each zone and distance travelled (cm) were analysed.

**Elevated Plus Maze (EPM).** Anxiety-like behaviour was assessed in the EPM [32] during a single 5 min session carried out 2 h after the OF. The EPM consisted of a black plastic structure with four arms (50 × 12 cm) forming a cross centred on a neutral square (12 × 12 cm). The maze was situated 50 cm above the floor, with two of the arms protected by vertical 40 cm high walls (closed arms), while the other two perpendicular arms had unprotected edges (open arms; adapted [33]). The anxiety ratio was measured based on the relative time the animal spent on the open arm.

**Spatial Object Recognition (SOR) in the Y maze.** SOR memory is a type of declarative or explicit memory, characterized as the ability to recognize whether different stimuli have been experienced before [34]. The protocol used for the two-trial recognition task to study memory in aged rats in the Y maze was adapted from Dellu et al. [35]. SOR in a Y maze includes a spatial component, location recognition, and direction of the arms, and is thus considered a hippocampal-dependent learning task [36] that also involves discrimination between two objects. The apparatus consisted of three black plastic arms (45 × 15 × 40 cm), and an equal preference object was placed at the end of each arm: a Lego-built inverted T shape (6.5 × 10.5 × 3.5 cm), or a soda can (11.5 × 7 cm). The sessions were recorded by camera (JVC, Everio Model GZ-X900) and analysed using tracking software (Smart Video Tracking System, Version 3, Panlab, Barcelona, Spain). Numerous distal visual cues (walls, air-conditioning machine, curtains and posters) were positioned around the Y maze to aid the animal’s orientation.

Twenty-four hours before the test, a habituation session was performed in which all the animals were allowed to explore the maze freely, without any object, during two 15 min trials and with a 30 min intertrial interval. The test phase consisted of two 15 min trials and an intertrial session of 30 min. In the first trial, two identical objects were placed at the end of the arms, whereas in the second trial, one object was the same as that used previously (familiar object), and a new object (novel object) was introduced. The familiar/novel objects and their locations were balanced between subjects. The exploration time of the familiar and novel object and the time exploring each arm were analysed.

#### 2.2.2. Protocols for Immunolabeling

**Sample acquisition.** Once the behavioural tasks were completed, a cohort of the animals was perfused intracardially with 4% paraformaldehyde (PFA) dissolved in phosphate-buffered saline (PBS, 0.1M [pH 7.4]) to fix the brain and maintain the tissue. The rat’s brain was extracted and post-fixed in fresh 4% PFA in PBS, and cryostat (40 μm thick) sections were then obtained, conserved in a glucose solution and stored at −20 °C. For each group of rats, representative and similar coronal brain sections containing the HPC were selected (≥3 sections per rat) to perform the different immunohistochemical analyses.

**Immunohistochemistry (IHC) of NeuN-positive neurons in the hilus of the DG.** Coronal brain sections were selected and stained for neuronal nuclear protein (NeuN), a marker of mature neurons (CR, n = 7; AL, n = 8; YA, n = 7). The tissue was first washed three times in Tris buffered saline (TBS [pH 7.6]) and then incubated for 15 min at room temperature (RT) in 0.9% H_2_O_2_ and 70% methanol in TBS to inactivate the endogenous peroxidases. The sections were then washed again successively in TBS-T (TBS + Triton X-100 [pH 7.6]), TBS and TBS-T, and blocked for 30 min with 10% new calf serum (NCS) diluted in TBS-T. The brain sections were then probed overnight at 4 ºC with a mouse anti-NeuN primary antibody (Mab 277: Chemicon, Millipore), diluted 1:1000 in NCS. The following day the sections were again washed successively in TBS-T, TBS and TBS-T, and then incubated for 1 h at RT with a biotin conjugated goat anti-mouse IgG (H + L) secondary antiserum (polyclonal SAB4600004: Sigma, Munich, Germany) diluted 1:1000 in 5% NCS/TBS-T, and then overnight at 4 °C. The next day, sections were incubated for 4.5 min at RT with SA-HRP (Perkin Elmer (Waltham, MA, USA), 1:3600) in TBS-T and with 3,3-diaminobenzidine as a substrate (DAB: Vector Labs SK-4100), in the presence of H_2_O_2_. After stopping the reaction and washing, the sections were mounted, dehydrated in ascending grades of ethanol (50–100%), cleared with xylene and cover-slipped in Petrex mounting medium. All the sections were visualized by bright field microscopy (Nikon Eclipse 80i) and analysed with ACT-1 software (Version 2.70: Nikon Corporation, Tokyo, Japan). Photos were taken with a 10X objective (four pictures per HPC from every section) under the same conditions of light, brightness and contrast, and the whole HPC was reconstructed from the images using the stitching plugin tool [37]. The polygon tool was then used to define the hilus, and the total area was measured in pixels and then translated into mm^2^ (1 pixel = 0.64 μm). ImageJ software (Version 1.53c: NIH) was used to manually quantify the number of NeuN+ cells in the hilus, dividing the numbers obtained by the total area measured to give a count per mm^2^, which was then averaged for each brain.

**Immunofluorescence for doublecortin-expressing (DCX+) cells.** An immunofluorescence assay was performed on brain tissue to visualize the immature DCX+ neurons in the HPC of the different experimental groups (CR, n = 8; AL, n = 8; YA, n = 7). Three sections per rat were first placed in a 24-well plate in PBS and maintained at 80 °C in 10 mM citrate buffer (pH 6) for 20 min to enhance antibody binding. The sections were then blocked for 45 min with 10% horse serum (HS) in 0.5% Triton X-100 (Sigma-Aldrich, St. Louis, MO, USA), after which they were probed for 48 h with the anti-DCX primary antiserum (ab18723, polyclonal rabbit IgG: Abcam, Spain), diluted 1:1000 in TBS-T, 1% HS and 0.1% sodium azide. After rinsing, the sections were incubated for 24 h with the secondary Alexa Fluor 488 Goat anti-Rabbit IgG antibody (H + L: Life Technologies, Carlsbad, CA, USA), diluted 1:1000 in TBS-T, 1% HS and 0.1% sodium azide to detect antibody binding to the DCX positive cells. The following day, the cell nuclei were then stained for 30 min with 4’,6-diamino-2- phenylindole (DAPI, 500 μL/well: Life Technologies, Carlsbad, CA, USA), diluted 1:1000 and finally, the sections were washed, mounted onto non-gelatinized glass slides and cover-slipped using Prolong Gold antifading reagent (Ref. P36930: Life Technologies, Carlsbad, CA, USA). The tissue sections were stored at 4 °C and then analysed under a fluorescent microscope (Eclipse 90i, Nikon) with a DXM 1200F digital camera (Nikon) attached, and the ACT-1 software (Version 2.70, Nikon Corporation) was used to analyse neurogenesis in four images per HPC region (CA1, CA2, CA3 and DG) with a Plan Apo 20X/0.75 DIC M/N2 objective, and employing the same conditions of light, brightness and contrast. To quantify the number of immature neurons in the HPC, the DCX+ cells were first counted manually in each HPC region using the same grid (0.85 inches^2^). Six dissectors for every grid were counted in the DG images, since this region was the only one to contain DCX+ cells. To obtain the density per mm^2^, the number of cells quantified were divided by the total area of the dissector (in mm^2^), and averaged for each brain and subject. Confocal microscopy (ZeissExaminer D1 AX10) with 20X and 40X immersion oil objectives was used to obtain a clearer vision of the DCX immunostaining, and to illustrate the results. Images were captured with confocal analysis software (ZEN2010) and analysed with Image J software (Version 1.53c; NIH).

**Immunofluorescence for Iba-1+ cells.** Iba-1+ cells were visualized in brain tissue by immunofluorescence to assess hippocampal neuroinflammation in the different experimental groups (CR, n = 7; AL, n = 6; YA, n = 5). Brain sections were subjected to antigen retrieval and blocking (1% HS), and were then probed for 48 h with a primary rabbit polyclonal antiserum against Iba-1 (IgG: Wako Pure Chemical Industries, Ltd., Osaka, Japan), and diluted 1:500 in TBS-T, 1% HS and 0.1% sodium azide. A secondary Alexa Fluor 555 goat anti-rabbit IgG antibody (Life Technologies; Carlsbad, CA, USA), diluted 1:1000 in TBS-T with 1% HS and 0.1% sodium azide, was used to detect antibody binding in the sections, which were then mounted on non-gelatinized glass slides using Prolong Gold antifading reagent (Life Technologies; Carlsbad, CA, USA; Ref. P36930). The brain sections were visualized under an Eclipse 90i microscope (Nikon), obtaining images with a digital camera (DXM 1299F Digital Camera, Nikon) controlled by the ACT-1 software (Version 2.70, Nikon Corporation). One general overview image per HPC was obtained with the Plan Apo 2X/0.1 objective in order to visualize and estimate its total area, and the ImageJ software was used to quantify the microglial cells in the CA1, CA2, CA3, CA4 and DG using an in-house plugin provided by the Institut de Neurociències (UAB).

### 2.3. Statistical Analysis

The IBM SPSS Statistics (v28) software (Chicago, IL, USA) was employed for the data analysis. The procedure employed for the comparisons was one-way analysis of variance (ANOVA). Despite the low sample size for the study, it must be highlighted that the current simulation analyses with Monte-Carlo modelling provide evidence for the robustness of the ANOVA even in the presence of heteroscedasticity and non-normal distributions [38]. In addition, the effect size for the global one-way F-test was measured using the partial eta-squared (η2) score, considering values of 0.06 as poor, 0.10 as mild and 0.25 as large [39]. The effect size for the pairwise comparisons was calculated with Cohen’s-*d* value (|*d*| < 0.20 was poor, |*d*| > 0.20 low, |*d*| > 0.50 mild, and |*d*| > 0.80 large [38]). Because of the small sample size and the lack of underpowered analysis, relevant differences for any statistical significance were considered to be (*p* ≤ 0.05) or an effect size at least in the mild range.

Pearson correlations were used to assess the association between a new variable, calculated as the ratio of the time (in seconds) exploring the novel versus the familiar object with the Iba-1 area (μm^2^), stratified based on the group condition. Due to the strong association between statistical significance for the R-coefficients and sample size (values interpreted as large effect size could achieve a non-significant result in small samples), |*R*| > 0.10 was interpreted as low effect size, |*R*| > 0.24 mild, and |*R*| > 0.37 large (these thresholds corresponding to a Cohen’s-d of 0.20, 0.50 and 0.80, respectively [40]).

## 3. Results

The results of the ANOVA analysis used to compare the mean scores registered by the animals in each group for the behavioural and the immunolabelling parameters are indicated in Table 1 and Table 2.

### 3.1. Behavioral Measures

**Figure 2:**
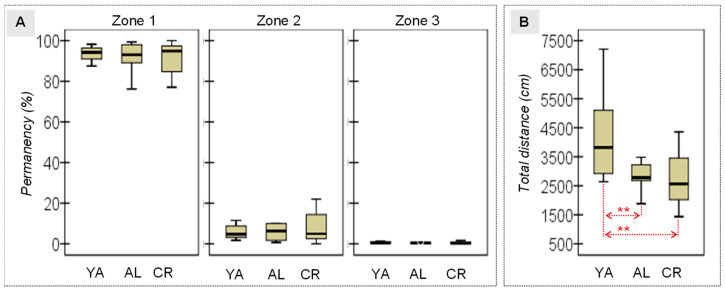
(**A**) Relative permanence in the three zones of the OF. (**B**) Locomotor activity (total distance travelled). YA, young adult; AL, ad libitum; CR, caloric restriction; ** Large–high effect size (|*d*| > 0.80).

**Open Field.** The ANOVA evidenced the lack of differences in terms of the time the rats remained in the different zones of the OF (Table 1 and Figure 2A), suggesting that neither aging nor diet influenced their levels of anxiety or their capacity for exploration. However, differences in their locomotor activity were detected when the distance travelled was considered and, specifically, a greater mean distance was recorded for the adult rats when compared to either of the older groups of rats (Figure 2B), suggesting that motor activity diminishes with age.

**Elevated Plus Maze (EPM).** Considering the relative time spent in the open arm during the EPM, the ANOVA confirmed that there were no significant differences between the groups in terms of the time they remained in the open arm, indicating comparable levels of anxiety-like behaviour in each group of animals (Table 1).

**Figure 3:**
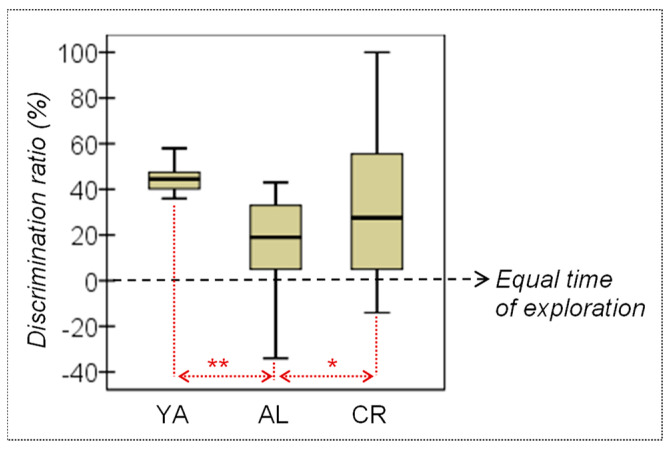
YA, young adult; AL, ad libitum; CR, caloric restriction. * Mild–moderate effect size (|*d*| > 0.50); ** Large–high effect size (|*d*| > 0.80).

**Spatial Object Recognition (SOR) in the Y maze.** SOR was evaluated through the discrimination ratio, which was calculated by comparing the time each group of rats spent (in seconds) exploring the novel versus the familiar object during the Y maze test (see Table 1 and Figure 3). No difference was found between the YA and CR rats, indicating that the animals in both these groups have a similar discrimination ratio. However, the AL animals recorded a lower mean discrimination ratio than both the YA and the CR rats. Regarding the discrimination ratio itself, since a value equal to 0 reflects rats exploring both objects for an equivalent amount of time, three one sample T-tests were performed to compare the mean ratio registered for each group against the fixed theoretical mean μ = 0, which represents a chance exploration time. Relevant results were observed particularly for the YA and the old CR animals, which apparently spent significantly more time exploring the novel object than the familiar object, whereas no significant differences were observed in the old AL group (YA *T* = 14.82, *df* = 11, *p* < 0.001, |*d*| = 4.28; AL *T* = 2.07, *df* = 8, *p* = 0.072, |*d*| = 0.69; CR *T* = 2.99, *df* = 11, *p* =0.012, |*d*| = 0.86). These results suggest that a CR regime can ameliorate age-related deficits in object recognition memory.

### 3.2. Neural Density in the Hilus of the DG

**Figure 4:**
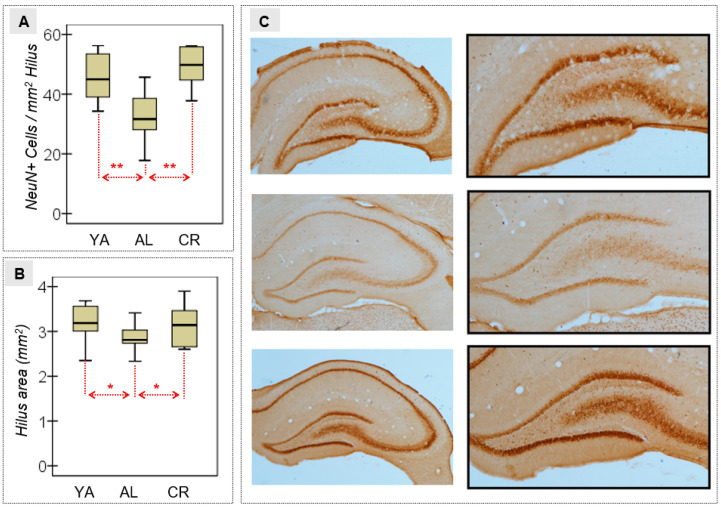
(**A**) Quantification of the neural density in the hilus (proportion of NeuN-positive cells over the hilus area). (**B**) Estimation of the hilus area for each experimental group measured in mm^2^. (**C**) NeuN immunostaining with DAB in the hilus. Representative images of the NeuN staining in the hippocampus (HPC) (brown) with high magnification images of the hilus (boxed). One section per animal from each group was selected to illustrate the data. Scale bars = 500 μm. YA, young adult (top); AL, ad libitum (middle); CR, caloric restriction (bottom); * Mild-–moderate effect size (|*d*| > 0.50); ** Large–high effect size (|*d*| > 0.80).

The ANOVA of the NeuN+ cells quantified in the hippocampal hilus was considered a measure of the effects of age and diet on neuronal density, and it revealed differences between the distinct groups of animals. As such, AL aged rats had a lower mean neuronal density than YA and CR rats (Table 2 and Figure 4A). Moreover, a small effect size was evident when the hilus area (mm^2^) was estimated (Figure 4B), with a lower mean in the AL group relative to the other two conditions. High magnification images of the HPC illustrated the variation in neuron density between the two experimental groups (Figure 4C).

### 3.3. Density of Immature Neurons in the DG

**Figure 5:**
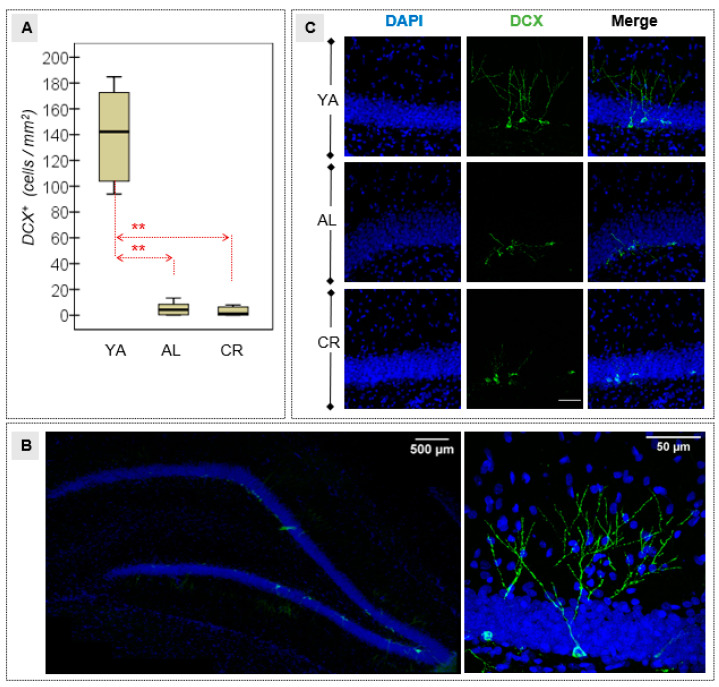
(**A**) Quantification of the immature neurons in the DG, as represented by the proportion of DCX+ cells in the hippocampal area. (**B**) DCX immunostaining in the DG of YA and older rats: a representative image of the hippocampal DG with a high magnification (inset) of a DCX+ cell from a YA animal. (**C**) Representative micrographs of DCX+ immature neurons in the DG (green) to illustrate the analysis, with the nuclei stained with DAPI (blue). Scale bar = 50 μm. YA, young adult (top); AL, ad libitum (middle); CR, caloric restriction (bottom); ** Large–high effect size (|*d*| > 0.80).

Differences were observed between the YA group and both the groups of old animals in terms of the number of neuroblasts or immature neurons detected in the DG through DCX immunofluorescence. Specifically, there were significantly fewer immature neurons in all aged rats (Table 2 and Figure 5A), suggesting that there is significantly less AHN in the older rats and that the CR regime did not significantly preserve neurogenesis in older animals. These results were illustrated with greater detail through confocal microscopy (Figure 5B), highlighting the structural changes in terms of the number of branches and the dendritic arborization in the three experimental groups (Figure 5C).

### 3.4. Microglial Activation in the HPC

**Figure 6:**
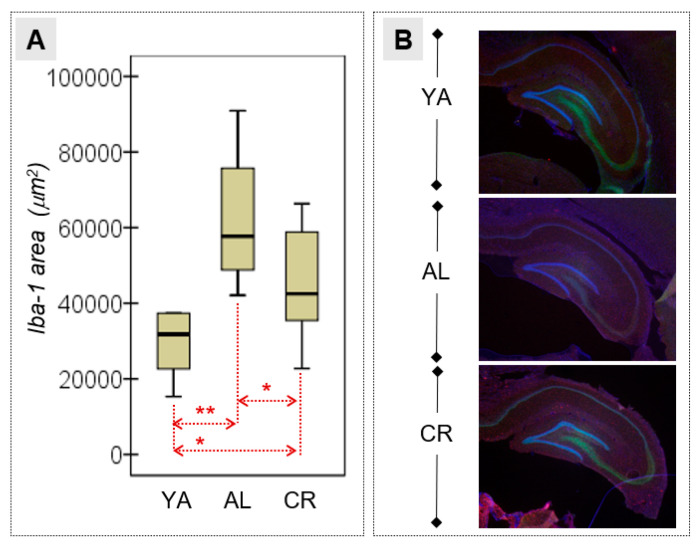
(**A**) Iba-1 expression in the HPC. (**B**) Hippocampal images showing Iba-1 expression in the three groups of animals: DAPI stains the cell nuclei blue, while the microglial cells are immunolabelled for Iba-1 in red. YA, young adults (top); AL, ad libitum (middle); CR, caloric restriction (bottom); * Mild–moderate effect size (|*d*| > 0.50); ** Large–high effect size (|*d*| > 0.80).

A quantitative immunohistochemical analysis of the area occupied by Iba-1+ cells (μm^2^) was performed to evaluate the extent of neuroinflammation in the HPC of the different groups of animals (Figure 6). Pairwise ANOVA comparisons highlighted the differences between the three groups, with the largest mean area registered in the AL group, followed by the CR and YA groups (Table 2). Hence, natural aging appears to influence neuroinflammation, and this phenomenon can be at least in part ameliorated by CR.

### 3.5. Correlation between Recognition Memory and Iba-1 Expression

**Figure 7:**
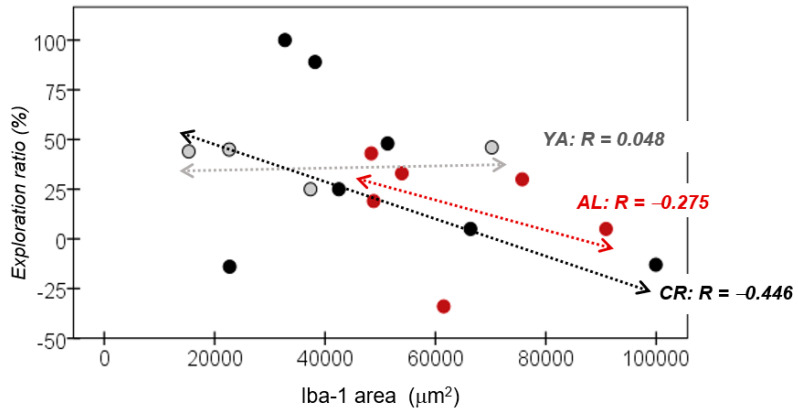
YA, young adult; AL, ad libitum; CR, caloric restriction.

No relevant correlation was found between the exploration ratio recorded in the Y maze and the level of Iba-1 expression in the HPC detected in the YA group (*R* = 0.048). Nevertheless, a relevant negative association was found between these parameters in both the AL (*R* = −0.275) and the CR groups (*R* = −0.446: Figure 7). This negative correlation suggests that the enhanced neuroinflammation evident in the old animals was associated with worse memory in the object recognition Y maze test.

## 4. Discussion

Non-pathological aging is linked to a deterioration in health and cognitive decline due to cellular, molecular and functional changes in humans and rodents. Mankind’s interest in combatting these changes has been seen throughout the ages and more recently, attempts to counteract this natural process have become the focus of considerable research [3]. Interestingly, reducing caloric intake has been proposed as a strategy to prevent the cognitive deterioration and loss of health associated with aging, and to delay the onset of neurodegenerative diseases [41]. The main aim of the present study was to characterize the effect of ageing and a low-caloric diet on spatial memory recognition, neuroinflammation, hippocampal neural loss and AHN. As a result, we provide evidence of the protective effects of CR against neuronal damage in the HPC of aged rats. CR enhances recognition memory in AL aged rats, which is associated with milder neuroinflammation and less neuronal loss in the HPC. However, in our study, administration of a low-caloric diet in rats from four months of age had no observable effect on neurogenesis.

Our findings indicate that a CR dietary intervention attenuates the age-related decline in SOR memory, as evaluated in the Y maze object recognition task. Because rodents have an innate preference for novelty, if the rat recognizes the familiar object, it will spend most of its time at the novel object [42]. Therefore, this behavioural task involves the ability to discriminate a familiar from a novel object and the capacity to form a cognitive map of the space, and it reflects the use of SOR memory [43]. Preference for the novel object, as witnessed by the increase in the time exploring that item, indicates that a memory trace for the familiar object was properly encoded, consolidated and then retrieved to guide the rodent’s behaviour during the test [44]. During aging, both object and spatial representations and the ability to detect changes in the environment deteriorates, such that the interaction with the novel object in the Y maze and spatial memory recognition is usually negatively affected [45,46,47,48], as also occurs in non-spatial object recognition tasks [49,50]. Accordingly, a meta-analysis found general differences between young and older adults in terms of recognition memory [51].

We observed here that CR moderately improves spatial memory recognition when measured in aged animals with a short-term delay (30 min), since the AL group but not CR animals displayed age-related difficulties in object recognition memory. This effect did not appear to be attributed to changes in spontaneous motor activity nor to anxiety-like behaviour, as no differences were evident between each group of animals in exploration within the OF or in the time spent in the open arms of the EPM. Interestingly, and similarly to our results, animals fed with a CR diet [52] or an antioxidant diet [53,54] have been seen to experience modest improvements in memory recognition. In addition, CR partially rescued recognition memory deficits in mouse models of neurodegeneration [55,56], although elsewhere controversial results were obtained, especially when memory is assessed in aged animals with shorter delays [57,58,59].

Although other brain structures may underlie recognition memory [60], the formation and recall of recognition memory have largely been attributed to the integrity of the HPC [61,62]. In this regard, regulated expression of molecular markers such as c-Fos has been observed during object recognition behavioural tasks [63]. On the other hand, lesions or chemical inactivation of the HPC impairs SOR recognition [64]. Interestingly, consuming a low-calorie diet has been proposed to protect neurons from age-dependent hippocampal behavioural impairment [65,66]. Indeed, CR was seen to ameliorate age-related memory and the CR employed exerted its neuroprotective effects by modulating astrocyte activity, which enhanced the neuronal density in the hippocampal hilus [65]. Moreover, a correlation between age-related decline in hippocampal-dependent memory tasks and a reduction in hippocampal volume during aging has been observed [2].

One of the mechanisms underlying brain dysfunction during aging is neuroinflammation [6,67,68]. Physiological aging is characterized by a chronic sub-clinical elevation of inflammatory markers [6], such as immune cells, cytokines or chemokines. In turn, these changes are critical in the regulation of neural stem cell survival, proliferation and maturation [69]. Here, we observed a detrimental effect of aging on hippocampal inflammatory responses since there was an increase in microglia as witnessed by the Iba-1 immunoreactivity in both groups of old animals relative to the younger adult rats. Interestingly, the dietary intervention had a significant, moderate mitigating effect on inflammatory expression, as the CR partially reversed neuroinflammation in the old animals. Other studies have also revealed a significant increase in hippocampal inflammation during aging [22,50,70], demonstrating that CR might attenuate markers of neuroinflammation and plasma cytokines [71]. This attenuation of microglial activation might dampen the release of pro-inflammatory cytokines, helping to prevent neural loss and the impairment of cognitive performance during aging [72,73]. In keeping with our results and those seen elsewhere, drugs that are considered to mimic CR could also exert their beneficial effects on health by regulating chronic neuroinflammation. For example, resveratrol, spermidine and metformin have all been shown to suppress pro-inflammatory responses, and to provide protection against inflammatory conditions in a range of animal models [6]. By contrast, it is known that exposure to hypercaloric diets induces a metabolic and proinflammatory state in brain regions such as the HPC, leading to a deterioration in memory recognition [74].

Both the effects of age in maintaining chronic inflammation and those of CR in decreasing it may contribute to both the survival and the proliferation of hippocampal neurons. In this regard, it is known that aging is associated with a significant non-linear decline in neurogenesis due to a reduction in the proliferating progenitors in vivo [8]. Moreover, failure to incorporate adult-born neurons into hippocampal networks compromises cell fate, leading to enhanced apoptosis and phagocytosis by microglia, endangering the brain’s ability to respond to age-dependent damage and augmenting cognitive decline [75]. Considering these factors and the results obtained here, the effect of CR may be two-fold. On the one hand, by dampening inflammation in the HPC, a low-caloric diet could alleviate age-associated neuronal death and in turn, may facilitate AHN. Accordingly, our findings corroborate the neuronal loss in the hippocampal hilus with age, given the significant differences in NeuN staining (a nuclear antigen of mature neurons) between the YA and AL groups. A decrease in neuronal density across the life-span has previously been demonstrated by measuring NeuN in the DG of aged female rats [50]. However, no a loss of hippocampal neurons was seen earlier in ageing rats [76], although by focusing on the hilus, a region that contains fewer neurons, we probably obtained a more consistent count here as it is easier to assess the whole area. More importantly, significant differences were detected here between the CR and AL groups, supporting the potential benefits of CR regimes in preserving hippocampal neurons during aging. These findings are consistent with other studies indicating that a CR diet preserves hippocampal neurons and synaptic plasticity during aging, as well as delaying the onset of neurodegeneration [77]. In terms of neurogenesis, our immunohistochemistry data indicate a marked decrease in the number of adult-born immature neurons in the DG of aged rats, in line with data presented elsewhere [50,78]. However, the dietary intervention did not prevent these effects since the number of DCX+ cells in the DG of both old CR and AL animals was significantly lower than in the YA rats.

The impact of CR on AHN remains unclear and somewhat controversial. While CR has been seen to increase the number of divisions that neural stem cells and NPCs undergo [8,14,79], this was not the case elsewhere, but rather CR was seen to enhance neurogenesis by promoting the survival of newly generated cells [80]. It is important to note that although CR may alleviate the decrease in NPC division in the aged female brain, this increase in the proliferative activity of stem cells and NPCs did not significantly augment the number of DCX+ new-born neurons [8,79]. Hence, the CR-induced increase in NPC proliferation is not fully translated into the generation of new mature neurons [79]. Importantly, while there are no clear gender differences in hippocampal neurogenesis in young adult mice [81], a CR-induced increase in the number of dividing cells has only been observed in females. Importantly, in aged animals, changes in hormonal levels may affect neurogenesis in males and females to a different extent [79].

In terms of neurogenesis, no significant increase in DCX+ neuroblasts was observed in old animals that followed a CR diet, suggesting that this regime does not increase neuron production. By contrast, CR may exert neuroprotection due to an increase in NPC proliferation, although, as proposed previously, this expansion may not fully translate into the generation of new mature neurons [79]. Such a response might reflect how aging not only reduces neurogenesis but also alters cell fate decisions, that is, the differentiation of new-born cells into neurons diminishes with age, since after exiting the cell cycle more cells adopt an astrocyte phenotype rather than a neuronal one [15]. When progenitor cells differentiate into astrocytes, they lose their ability to divide and generate new neurons. This may be linked to the loss of hippocampal neural stem cells, with a depletion of neurons and more astrocytes produced with age [14,15,79]. Together, the data presented here do not indicate any increase in neuroblast density, suggesting that CR may offer a neuroprotective effect rather than promoting neurogenesis. Consequently, further studies will be required to determine whether or not these new-born cells survive and mature into functionally integrated neurons [8,14,79,80,82].

To our knowledge, this is the first study to report the effects of CR on SOR memory that also evaluated the possible mechanisms underlying neural loss in the hilus, neurogenesis in the DG and the accumulation of microglia in the HPC in 24-month-old rats. Our main results indicate that the pro-cognitive effects of a CR diet employed from 4 to 24 months of age in male Wistar rats could be related to less neuroinflammation and consequently, may be due to enhanced protection of hippocampal neurons. Taken together, our major finding supports the beneficial effects of a CR diet to slow-down age-related structural and functional changes in the hippocampal formation.

## 5. Conclusions

This study provides evidence that a CR intervention ameliorates the effects of aging on SOR memories in a Y maze task, in male rats. In addition, the data obtained offers insight into the physiological mechanisms underlying the benefits of CR in hippocampal plasticity, specifically by evaluating how CR diminishes hippocampal neuronal loss as well as dampening proinflammatory activity. Future research into the contribution of neuroinflammation on age-related cognitive deficits and the benefits of a CR diet in preventing these changes will be of great interest to fully understand the specific mechanisms driven by events in this intervention.

## Figures and Tables

**Table 1:** Behavioural results obtained and assessed by one-way ANOVA.

	YA		AL		CR		Factor Group				YA vs. AL		YA vs. CR		AL vs. CR	
	Mean	SD	Mean	SD	Mean	SD	*F*	*df*	*p*	η2	*p*	*|d|*	*P*	*|d|*	*p*	*|d|*
1 Behavioral measures																
OF: Time zone-1 (%)	92.09	7.91	89.95	10.66	89.33	13.03	0.21	2/31	0.809	0.014	0.654	0.23	0.535	0.26	0.898	0.05
OF: Time zone-2 (%)	7.09	7.01	9.58	10.46	10.21	12.55	0.31	2/31	0.739	0.020	0.585	0.28	0.461	0.31	0.891	0.05
OF: Time zone-3 (%)	0.83	1.03	0.47	0.43	0.46	0.53	0.92	2/31	0.410	0.058	0.285	0.45	0.232	0.45	0.967	0.03
OF: Distance (cm)	4224.7	1557.4	2965.6	711.9	2726.9	916.8	5.69	2/31	0.008 *	**0.275** **†**	0.019 *	**1.04** **†**	0.003 *	**1.17** **†**	0.643	0.29
EPM: Time open arm (%)	19.44	15.01	23.56	20.30	33.50	25.72	1.43	2/31	0.256	0.087	0.595	0.23	0.127	0.67 †	0.321	0.43
SOR: Discriminat.ratio (%)	44.90	10.50	16.22	23.51	32.25	37.41	3.02	2/31	0.064	**0.167** **†**	0.020 *	**1.58** **†**	0.251	0.46	0.180	**0.51** **†**

Note: OF, open field; EPM, elevated plus maze; SOR, spatial object recognition; discrimination ratio   = [novel object contact (s)  −  familiar object contact (s)]/total object contact time (s)]_;_ AL, ad Libitum; CR, caloric restriction; YA, young adult; SD, standard deviation; df, degrees of freedom; η2, Eta-squared; |*d*|, Cohen’s-*d*; * Bold, significant parameter (0.05 level); † Bold, effect size within the mild-moderate to high-large ranges. 1 Sample sizes: YA = 12, AL = 10, CR = 12.

**Table 2:** Assessment of the immunolabelling data evaluated by one-way ANOVA.

	YA		AL		CR		Factor Group				YA vs. AL		YA vs. CR		AL vs. CR	
	Mean	SD	Mean	SD	Mean	SD	*F*	*df*	*p*	η2	*p*	*|d|*	*p*	*|d|*	*p*	*|d|*
1 Neural density																
NeuN+ (cells/mm^2^)	45.50	8.62	32.66	9.89	48.99	7.54	6.29	2/16	0.010 *	**0.440** **†**	0.019 *	**1.38** **†**	0.503	0.43	**0.004**	**1.86** **†**
Hilus area (mm^2^)	3.16	0.48	2.87	0.35	3.15	0.50	0.94	2/16	0.409	**0.106** **†**	0.248	**0.70** **†**	0.964	0.02	0.267	**0.66** **†**
2 Immature neurons																
NeuroDCX	139.18	39.17	4.93	5.01	2.94	3.47	94.10	2/20	0.001 *	**0.904** **†**	0.001 *	**4.81** **†**	0.001 *	**4.90** **†**	0.856	0.46
3 Microglial activation																
Iba-1 area (μm^2^)	35,458.8	21,180.5	63,205.4	16,976.0	50,533.2	25,811.3	2.18	2/15	0.148	**0.225** **†**	0.054	**1.45** **†**	0.259	**0.64** **†**	0.316	**0.58** **†**

Note: 1 Sample sizes: YA = 6, AL = 7, CR = 6; 2 Sample sizes: YA = 7, AL = 8, CR = 8. 3 Sample sizes: YA = 5, AL = 6, CR = 7. * Bold, significant parameter (0.05 level); † Bold, effect size within the mild-moderate to high-large ranges.

## Data Availability

Individuals may contact Gemma Guillazo regarding the availability of the data, as there are studies ongoing using this data. To avoid overlapping research efforts, Guillazo will consider requests on a case-by-case basis.
